# Effect of Acute Myocardial Infarction on a Disintegrin and Metalloprotease with Thrombospondin Motif 13 and Von Willebrand Factor and Their Relationship with Markers of Inflammation

**DOI:** 10.1155/2020/4981092

**Published:** 2020-02-11

**Authors:** Abeer A. Al-Masri, Syed Shahid Habib, Ahmad Hersi, Hana Al Zamil

**Affiliations:** ^1^Department of Physiology, College of Medicine, King Khalid University Hospital, King Saud University, Riyadh, Saudi Arabia; ^2^Department of Cardiac Sciences, College of Medicine, King Khalid University Hospital, King Saud University, Riyadh, Saudi Arabia

## Abstract

**Objectives:**

Coagulation mechanisms and fibrinolytic assembly are important components role players of acute myocardial infarction (AMI) progression. Our study objective was to see the serial variations in the levels of Von Willebrand factor (VWF) and A Disintegrin and Metalloprotease with ThromboSpondin motif (ADAMTS13) over the course of AMI and to determine their relationship with the cardiovascular risk markers and the patient's clinical characteristics.

**Methods:**

This project was done at the departments of Emergency Medicine, Physiology and Cardiac sciences of King Saud University Medical City. We studied ADAMTS13, VWF, fibrinogen, and CRP levels in 80 patients with AMI when patients were admitted; post AMI by 3-4 days and at follow-up of 3 months. We compared them with a control group consisting of 36 subjects.

**Results:**

AMI had significantly lower levels of ADAMTS13 at AMI and after 3-4 days; at follow-up the difference in levels was nonsignificant, when compared with controls. Similarly, VWF levels were significantly higher in AMI and remained high even at follow-up compared to control subjects. VWF/ADAMTS13 ratio was also significantly higher at AMI and 3-4 days while at follow-up difference was nonsignificant compared to control subjects. Regression analysis between hsCRP and ADAMTS13 showed an inverse relationship (*r* = 0.376, *p* < 0.01), while correlation with VWF was significantly positive (*r* = 0.376, *p* < 0.01), while correlation with VWF was significantly positive (

**Conclusions:**

Increased levels of VWF and reduced levels of ADAMTS13 activity may contribute to the pathogenesis of acute myocardial infarction and might prove to be important mediators of AMI progression.

## 1. Introduction

Coagulation assembly and its fibrinolytic components are the leading cause of mortality worldwide and has estimated management burden of higher than $250 billion that is expected to be tripled by the year 2030 [1]. In addition management of thrombotic and haemorrhagic attacks may add to increase incidence and cost of management of coagulation disorders [2].

The vascular endothelium is a primary source of several very essential agents that participate in cardiovascular pathophysiology. The VWF is synthesised in endothelial cells and is then stored. It mediates platelet aggregation and adhesion [3]. It carries factor VIII in circulation and mediates initial platelets that stick to the sub endothelium following vascular endothelial damage as a result of hyperlipidaemia, smoking, or hypertension [4, 5].

Several factors can affect the levels and function of VWF like blood group, inflammatory state, and proteolytic activity of ADAMTS13 [6]. Tyrosine-842 and methionine-843 bonding is cleaved by ADAMTS13 in the VWF multimers domain A2, resulting in two 140 and 176 kDa portions. The 2 fragments behave less actively in aggregation of platelets than the uncleaved VWF multimers [7, 8].

Different studies suggested that high VWF levels cause endothelium dysfunction and damage caused to it can be an important thrombotic and atherosclerotic marker. A recent meta-analysis reported that plasma VWF was significantly increased after onset of AMI until 24 h and persisted high for one week. The levels were reduced to normal at fourteenth day after AMI. They suggested that the changing kinetics of plasma VWF after AMI might provide a new insight in monitoring AMI progression [9]. The highest VWF quartile has been reported to predict the highest risk for fatal and nonfatal CV events and has independent prognostic value compared to intracellular adhesion molecules [10]. In a very recent report the functional effect of the VWF C4 domain for VWF-mediated platelet aggregation was explored and showed that it acted in a shear-dependent manner to lead to AMI. This provided the first ever evidence that a functional variant of VWF is playing a role in arterial thromboembolism [11]. Another study demonstrated that higher VWF/ADAMTS13 ratio may be a significant predictor for adverse cardiovascular episodes after AMI [12]. Therefore, we aimed to examine serial changes in plasma ADAMTS13 antigen and VWF levels after AMI progression and to determine their relationship with the cardiovascular risk markers and the patient's clinical characteristics.

## 2. Methods

### 2.1. Study Design and Settings

This study project was conducted at the department Emergency, Physiology, and Cardiac sciences of King Saud University Medical city (KSUMC). It is a prospective study. This study was approved by institutional review board of KSUMC. Inclusion criteria were adult patients of already diagnosed with AMI according to American heart association, guidelines and we used standard criteria as reported [13]. Furthermore, individuals with concomitant systemic diseases and critically illness, recent infections, and surgical procedure in past 12 weeks were excluded.

We studied ADAMTS13, VWF, fibrinogen, and CRP levels in 80 patients with AMI during different time frames; when patients were admitted, post AMI by 3-4 days and at follow-up of 3 months. We compared them with a control group consisting of 36 subjects who were matched for gender, age, and body mass index (BMI). They were free of any clinical manifestations of coronary, peripheral or cerebral artery disease by detailed relevant history, clinical examination, and had normal electrocardiographic findings.

### 2.2. Analytical Details and Assays

Overnight fasting Venous blood samples were collected and analysed for blood lipids including serum total cholesterol (TC), serum High density lipoprotein (HDL), serum Triglycerides (TG), and serum Low density Lipoprotein (LDL) by colorimetry enzymatic assay, (Dimension, USA). hsCRP assays were done by turbidimetry (Quantex hsCRP ultra sensitive kits, by BIOKIT Spain). American Heart Association has published guidelines for measurement, evaluation, and risk levels of hsCRP which were used in our study [14]. ADAMTS13 levels in plasma were estimated by sandwich ELISA Human ADAMTS13 ELISA kits by American Diagnostica Inc., USA.

#### 2.2.1. Statistical Analysis

For data entry and analysis Statistical Package for Social Sciences (SPSS) version 20.0, was used. Descriptive characteristics and the lipid profile of the study patients were expressed as Mean ± SD (Standard Deviation). Kolmogorov–Smirnov^a^ and Shapiro–Wilk tests were used to see that data is following normal distribution or not. Those parameters which were not following normal distribution were analysed by nonparametric Mann Whitney test. For multiple group comparisons ANOVA was used for normally distributed data and Kruskal Wallis test for skewed data. Spearman's correlations were determined between Gensini score of CAD severity, vessel scores, hsCRP and lipids. *p*-value of <0.05 was taken as significant.

## 3. Results

**Figure 1:**
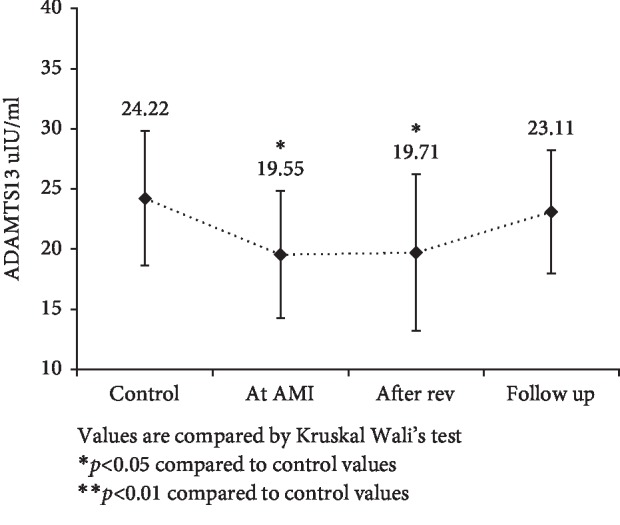
Comparison of ADAMTS13 levels in control, at AMI, after revascularization and at 4–6 weeks of follow-up in AMI patients with control subjects. Values are compared by Kruskal Wallis test. ^∗^*p* < 0.05 compared to control values. ^∗∗^*p* < 0.01 compared to control values.

**Figure 2:**
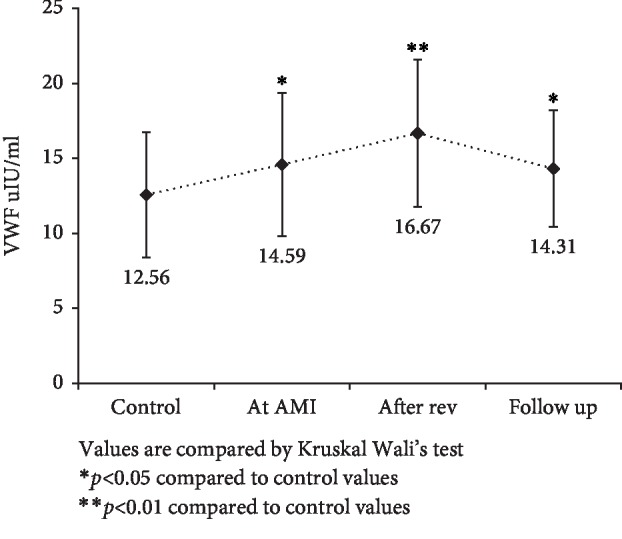
Comparison of VWF levels in control, at AMI, after revascularization and at 4–6 weeks of follow-up in AMI patients with control subjects. Values are compared by Kruskal Wallis test. ^∗^*p* < 0.05 compared to control values. ^∗∗^*p* < 0.01 compared to control values.

**Figure 3:**
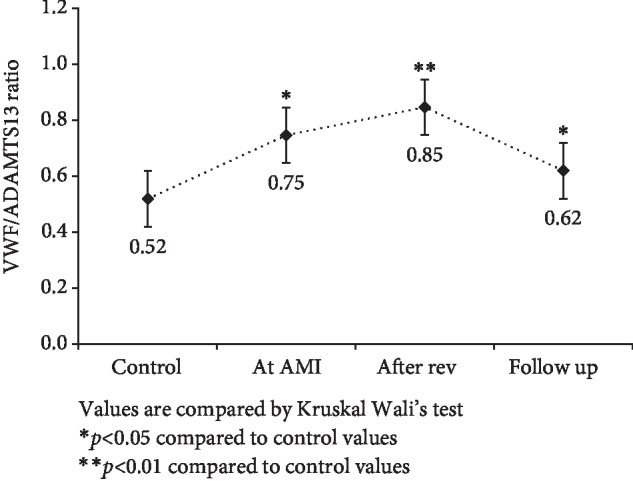
Comparison of VWF/ADAMTS13 ratio in control, at AMI, after revascularization and at 4–6 weeks of follow-up in AMI patients with control subjects. Values are compared by Kruskal Wallis test. ^∗^*p* < 0.05 compared to control values. ^∗∗^*p* < 0.01 compared to control values.

Patients with AMI showed significantly higher levels of fibrinogen and VWF levels compared to the control group (*p* < 0.01) as shown in Table 1. ADAMTS13 levels were significantly lower at AMI and after 3-4 days, while at follow-up levels the difference was nonsignificant (Figure 1). VWF levels significantly increased after AMI and remained high even at follow-up compared to control subjects (Figure 2). VWF/ADAMTS13 ratio was also significantly higher at AMI and 3-4 days while at follow-up difference was nonsignificant compared to control subjects (Figure 3). Spearman's correlations were determined between Gensini score of CAD severity, vessel scores, hsCRP and lipids. *p*-value of <0.05 was taken as significant.  Table 2 shows correlation analysis of ADAMTS13, VWF and VWF/ADAMTS13 ratio with demographic characteristics, lipid profile, Gensini score of CAD severity, vessel scores and hsCRP. It was observed that BMI (*r* = .287, *p* < 0.05) correlated positively with ADAMTS13. LDL correlated negatively with ADAMTS13 (*r* = −.333, *p* < 0.05), while it correlated positively with VWF (*r* = .374, *p* < 0.01) and VWF/ADAMTS13 ratio (*r* = .296, *p* < 0.01). Triglycerides correlated negatively with VWF (*r* = −.291, *p* < 0.05) and VWF/ADAMTS13 ratio (*r* = −.256, *p* < 0.05). HDL levels correlated negatively with VWF (*r* = −.297, *p* < 0.05). The levels of hsCRP correlated positively with VWF/ADAMTS13 ratio (*r* = .340, *p* < 0.01). All other correlations were not significant.

## 4. Discussion

In this study, we are reporting serial variations in VWF and ADAMTS13 after AMI and as it progresses. Moreover, their relationship between the plasma titers and the patient's clinical data. Compared to control subjects patients with AMI had significant changes in levels of both ADAMTS13 and VWF, even with their VWF/ADAMTS13 ratio titers at AMI, after 3-4 days and at follow-up. In a previously published work results showed inverse correlation between ADAMTS-13 and VWF, although, sample size was relatively small yet the effect was more significant after acute release of VWF. The same trend was observed in our study also [15]. Crawley JTB et al. have shown positive correlation of VWF with fibrinogen, showing that it may behave as acute-phase reactant. In our study, there was negative associations between ADAMTS13 and hsCRP [16].

An interesting recent study has reported reduced ADAMTS13 in patients with AMI and they used data from samples taken on hospital admission. Consequently, whether reduced ADAMTS13 was a cause or effect of MI in those individuals could not be determined. Our study gives an evidence that it may be the cause of AMI because at follow-up levels it became normal [17]. In line with our study it is also reported that decrease in ADAMTS13 and increase in titers of VWF lead to a higher risk of ischemia induced stroke and AMI. Oral contraceptives further increased this risk in women [18]. Moreover, we have reported recently that lower titers of ADAMTS13 have been associated with a higher mortality in septic shock patients. It was a better comparable predictor to the well-known standard score of Acute Physiology and Chronic Health Evaluation II (APACHE II) for prediction of mortality [19].

The immediate rise in VWF in acute coronary syndrome (ACS) is associated with adverse outcomes [20, 21]. In a prospective longitudinal observational study by Green D et al., it was found that 10 months prior to the heart attacks, median values for VWF and the ratio of VWF and ADAMTS13 were higher in cases than in controls. And there was a significant trend toward an increase in the ratio prior to a cardiovascular event [22]. Masakazu et al., revealed that platelet thrombus formation is the result of a derangement between ADAMTS13 balance and VWF in plasma, and also revealed a pivotal role of ADAMTS13 in platelet stickiness. It is regulated through its inhibitory effect of abnormally large multimers of VWF during normal flowing states. VWF antigen/ADAMTS13 ratio has a significant prognostic power at different time points after heart attacks concomitant with higher chances of adverse outcomes in clinical practise, like heart failure and thrombosis [23].

In unstable angina (UA) patients with the tendency of thrombogenicity increases if the proportion between VWF and ADAMTS13 changes significantly. In unstable angina patients ADAMTS13 were lower compared with stable angina and showed an inverse correlation between ADAMTS13 and VWF. All these observations are consistent with our results in AMI patients. Similar to our results the antigen levels after about six months follow-up became nonsignificant when compared with UA patients in acute phase [24]. The limitations of our study are a relatively small number of samples and short duration. Long term prospective trials at large scale are required to explore the predictive role of VWF and ADAMTS13 in cardiovascular diseases.

## 5. Conclusions

Our data suggest that increased levels of VWF and reduced levels of ADAMTS13 activity may contribute to the pathogenesis of acute myocardial infarction. These markers might provide a new insight in monitoring progression of AMI.

## Figures and Tables

**Table 1:** Clinical and demographic data of control and patients with AMI.

	Control	AMI	
	*N* = 36	*N* = 50	
M/F	20/16	32/18	*p* values
Age	52.40 ± 8.62	54.83 ± 12.72	0.2282
BMI	26.41 ± 3.58	28.27 ± 6.21	0.2645
SBP mmHg	126.27 ± 3.25	131.97 ± 20.75	0.1170
DBP mmHg	75.35 ± 2.13	76.87 ± 14.65	0.5416
Fibrinogen	329.32 ± 60.72	425.09 ± 148.72^∗^	0.0383
VWF *µ*IU/L	12.56 ± 4.18	14.31 ± 3.88^∗^	0.0435
ADAMTS13 *µ*IU/L	24.22 ± 5.59	23.11 ± 5.11	0.5699
hsCRP mg/dl	0.28 ± 0.32	0.64 ± 0.75^∗∗^	0.0051
TC mmol/L	4.48 ± 0.60	4.37 ± 1.32	0.7492
TG mmol/L	1.11 ± 0.49	1.78 ± 1.03^∗^	0.012
LDL mmol/L	2.76 ± 0.53	2.82 ± 1.11	0.8423
HDL mmol/L	1.07 ± 0.32	0.75 ± 0.23^∗^	0.0012

Data is expressed as Mean ± SD. Systolic blood pressure (SBP), Diastolic Blood pressure (DBP), Differences were studied by Mann Whitney test for Fibrinogen, VWF, ADAMTS13 and hsCRP while Independent Student's *t* test for other parameters. Reference values for blood analytes: TC: <5.1 mmol/L, TG: >1.7 mmol/L, LDL: <2.5 mmol/L, HDL: 1.5 mmol/L, Fibrinogen: 150–400 mg/dl, hsCRP mg/dl: (Low <.1, Average .1–.3, High >.3).

**Table 2:** Correlation coefficients of anthropometric and metabolic characteristics of the subjects with VWF and ADAMTS13 levels in AMI patients.

	ADAMTS13	VWF	VWF/ADAMTS13 ratio
Age	−.056	.063	.048
BMI	.287^∗^	−.202	−.167
SBP	−.085	.169	.017
DBP	−.100	.138	.104
TC	−.237	.099	.133
TG	.179	−.291^∗^	−.256^∗^
LDL	−.333^∗^	.374^*^	.296^∗^
HDL	.025	−.297^∗^	−.239
Vessel score	−.046	.175	.070
Gensini score	−.080	.076	.074
hsCRP	−.230	.124	.340^∗∗^
Fibrinogen	−.204	−.017	−.042

Total cholesterol (TC), Triglycerides (TG), Low density Lipoprotein (LDL) and High density lipoprotein (HDL). ^∗^Correlation is significant at the 0.05 level (2-tailed). ^∗∗^Correlation is significant at the 0.01 level (2-tailed).

## Data Availability

The data used to support the findings of this study are available from the corresponding author upon request both in excel and SPSS format.
